# A bibliometric analysis on delirium in intensive care unit from 2013–2023

**DOI:** 10.3389/fneur.2025.1469725

**Published:** 2025-02-12

**Authors:** Xin Ma, Qingya Wu, Yue Ran, Xueqin Cao, Hua Zheng

**Affiliations:** ^1^Department of Anesthesiology, Hubei Key Laboratory of Geriatric Anesthesia and Perioperative Brain Health, and Wuhan Clinical Research Center for Geriatric Anesthesia, Tongji Hospital, Tongji Medical College, Huazhong University of Science and Technology, Wuhan, China; ^2^Department of Anesthesiology, Shanxi Bethune Hospital, Shanxi Academy of Medical Sciences, Third Hospital of Shanxi Medical University, Tongji Shanxi Hospital, Taiyuan, China

**Keywords:** delirium, intensive care unit, ICU, Citespace, VOSviewer

## Abstract

**Background:**

Delirium is a common manifestation of acute brain dysfunction among patients in the Intensive Care Unit (ICU), afflicting an estimated 30–35% of this vulnerable population. The prevalence of delirium in ICU settings has catalyzed a surge in academic interest, as evidenced by a growing body of literature on the subject. This study seeks to synthesize the progress in understanding ICU delirium through a bibliometric analysis.

**Methods:**

We conducted a comprehensive search of the Web of Science Core (WOS) Collection database for literature on ICU delirium, focusing on studies published between 2013 and 2023. Our analysis utilized two bibliometric software tools, Citespace and VOSviewer, to scrutinize the data across various dimensions, including country contributions, authorship patterns, publishing journals, key thematic terms, and other pertinent metrics, with the aim of identifying emerging trends in the field.

**Results:**

Our search yielded a total of 1,178 publications on ICU delirium within the WOS database from January 2013 to June 2023. The United States emerged as the leading contributor in terms of published articles, with Ely, E. Wesley being the most prolific author, having published 85 articles, and “Critical Care Medicine” as the journal with the highest number of publications, totaling 105. The application of literature clustering and keyword analysis revealed that future research is poised to delve deeper into areas such as pediatric delirium, risk factors, and the development of preventive and therapeutic strategies.

**Conclusion:**

This study employs bibliometric analysis to provide a multifaceted overview of the ICU delirium research landscape over the past decade. By examining the topic from various perspectives, we have not only mapped the current state of ICU delirium research but also illuminated potential avenues for future inquiry and areas of emphasis.

## 1 Introduction

Delirium, a brief condition of mental disorientation and cognitive impairment, is characterized by signs of inattention and disordered thought patterns (1). Delirium can be precipitated by factors, such as advanced age, cardiac surgery, history of depression or stroke, physical restraints, mechanical ventilation, and physiological internal environmental dysfunction (2, 3). It is prevalent in the ICU, and in extreme cases, it can be fatal (4). Due to a number of risk factors that are present in critically sick patients, the incidence rate of delirium in the ICU ranges from 45 to 87% (5–8). There is evidence suggesting that delirium in the ICU is associated with prolonged hospital stays, increased hospitalization costs, and higher rates of morbidity and mortality (9–14). However, the rate of missed diagnosis of delirium in clinical practice remains as high as 55% ~ 60% (1), and no effective drug has been available to prevent and treat ICU delirium.

This essay aims to explore the representation of delirium in the ICU over the last ten years utilizing Citespace and VOSviewer software, to examine the impact, hotspots, and trends of relevant literature. Citespace, a free JAVA application developed by Professor Chaomei Chen in 2004, provides bibliometric analysis and visualization (15). VOS is another software program developed specifically for scientific mapping and bibliometric visualization (16). By employing these tools, researchers are able to effortlessly review the literature and quickly locate relevant publications (17). This not only aids in understanding the current trends and patterns within the research on ICU delirium but also facilitates the effective utilization of classic articles in future investigative efforts.

## 2 Materials and methods

### 2.1 Data sources and search strategy

An investigation was conducted on the WOS database using the search term “delirium” AND (“intensive care unit” OR “ICU”). The search parameters were set as follows: the literature type was limited to “article,” the language was restricted to English, the time frame was from January 2013 to June 2023, and the index was specified as the Science Citation Index Expanded (SCI-E) within the Web of Science Core Collection. This collection encompasses a broad spectrum of high-quality scholarly literature and can serve as a more authoritative and representative data source. During the search process, we initially excluded documents irrelevant to the research topic. Subsequently, professional document management software (such as EndNote) was utilized to eliminate duplicate document records, ensuring that each document was counted only once in the analysis to guarantee data accuracy and reliability. In total, 1,178 articles were identified and included in the analysis. For further analysis, the final records were downloaded in .txt format and imported into visualization and bibliometric tools. The institution ranking was provided by Scimago Institutions Rankings (https://www.scimagoir.com/rankings.php), while the H-index for countries/regions was furnished by Scimago Journal and Country Rank (https://www.scimagojr.com/journalrank.php). The H-index represents the highest number of papers (H) that have been cited at least H times.

### 2.2 Data analysis

Bibliometric analysis was conducted using CiteSpace (https://citespace.podia.com/), covering aspects such as country, institution, author, keyword, category, reference, and journal. Time slicing (2013–2023), node types, and selection criteria (top 50 levels of most-cited or frequently occurring items) were included in the parameter settings.

CiteSpace generates knowledge maps composed of linkages and nodes. On these maps, the size of a node represents the quantity of articles, and the citation rings on the nodes denote the publication years. The degree of collaboration among nodes is manifested by the strength of the connections between them (18). The presence of red rings around a node signifies its high inter-node centrality and robust citation burstiness, and a node exhibiting both strong centrality and burstiness will yield a high sigma score (19). Keyword and author co-occurrences were visualized using VOSviewer software (version 1.6.18; https://www.vosviewer.com/). It distinguishes between research disciplines and themes using distinct colors and node sizes, making visual analysis of interdisciplinary research more intuitive and effective. In the co-occurrence map, larger nodes imply a higher frequency co-occurrence of the item. Origin 8.0 software was used to analyze the published data.

## 3 Results

### 3.1 Publications and years

**Figure 1:**
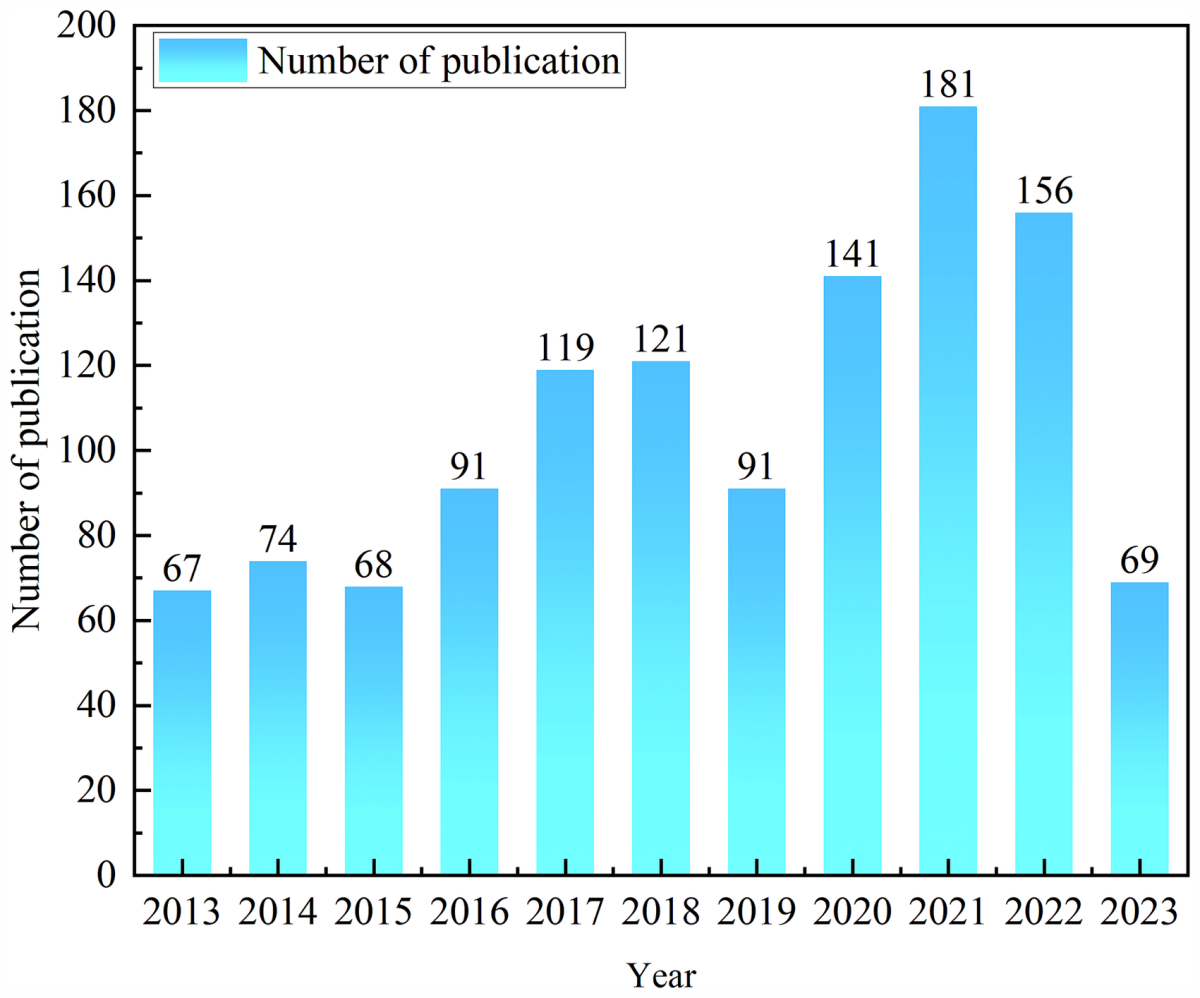
Trends in publications on delirium in the ICU.

The evolution of ICU delirium-related literature papers in the WOS database from January 2013 to June 2023 is depicted in Figure 1. The results clearly demonstrate that the number of publications related to ICU delirium exhibits a steady increasing trend year by year. Significantly more publications emerged in 2021 compared to any other year, indicating the heightened level of research activity in this subject during that period. However, the figure also reveals that the number of publications dropped dramatically in 2019. The primary reason for this outcome might be that the COVID-19 pandemic hampered the research efforts, such as the cancelation or postponement of international academic conferences and the interruption of research projects. By analyzing the disparities in column height across years, we can visually assess the increasing or decreasing trend of research enthusiasm in this subject. This provides us with a reference for predicting the future direction of research.

### 3.2 Authors

**Figure 2:**
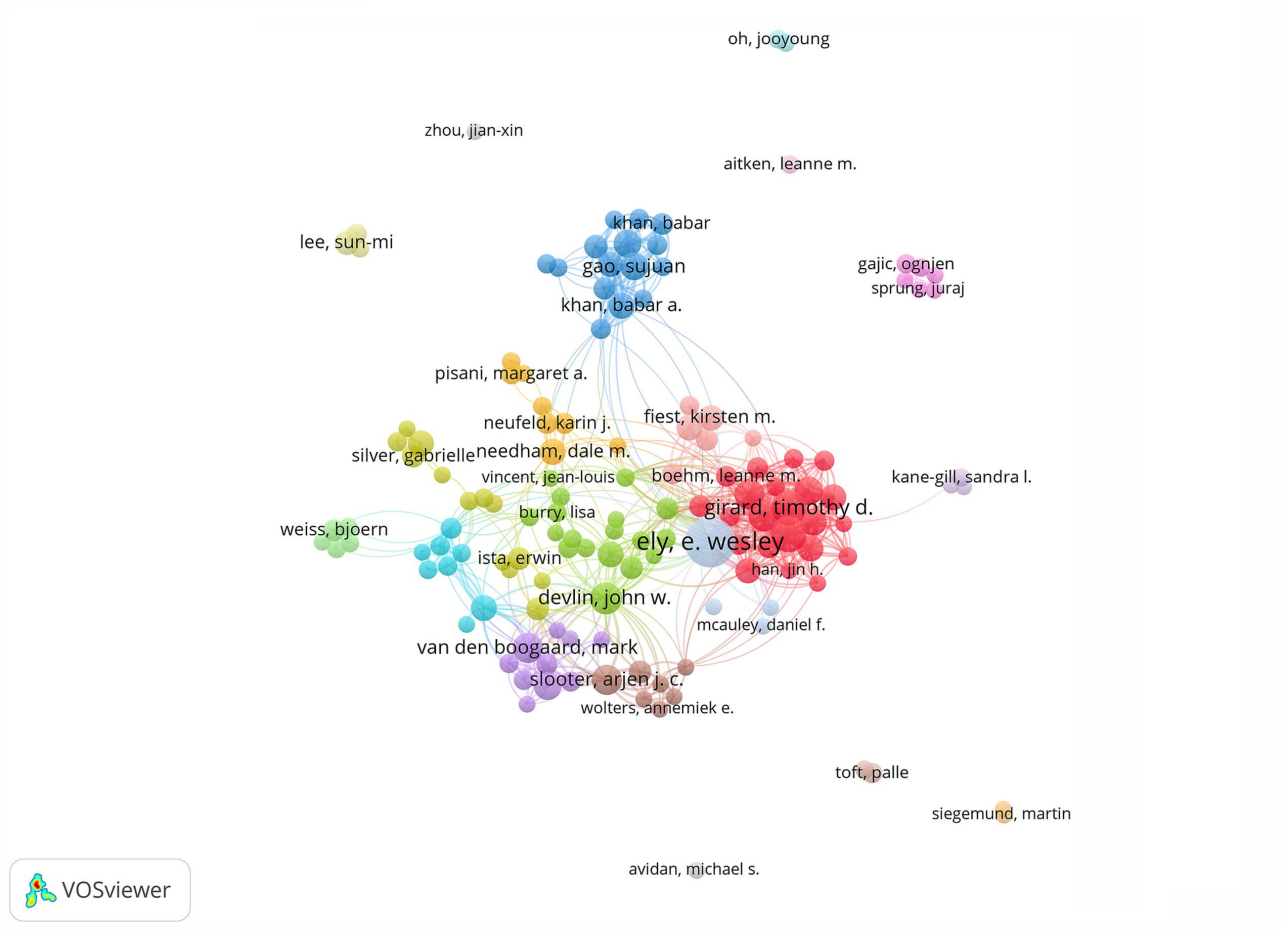
Visual map showing collaborations among authors. Each node symbolizes an individual author. The node’s size is scaled to reflect the volume of their published works, with larger nodes indicating a more prolific publication record. The connecting lines between nodes denote collaborative relationships, illustrating the interdisciplinary and cooperative nature of the research community.

Utilizing the VOSviewer software, we analyzed the authors and generated the author collaboration graph (Figure 2). Each node in the graph represents a different author. Τhe size of the nodes indicates the number of publications, and the proximity of the nodes indicates the degree of collaboration. Furthermore, the thickness of the connecting lines indicates the level of cooperation. According to the node size analysis, it is evident that Ely, E. Wesley is the author with the most publications (*n* = 85) and citations (6763). He also had a close cooperation with other authors. Meanwhile, the map shows that there are more than ten groups focus on ICU delirium, and the authors cooperated with each other both within and between groups. The top ten authors by the quantity of publications are listed in Table 1. Ely, E. Wesley, the most prolific author, has partnered with multidisciplinary experts from the ICU, psychology, and nursing to present a complete analysis of delirium in the ICU from different perspectives. In addition to helping international health organizations recognize the significance of ICU delirium, their work has aided in the creation and use of more precise and trustworthy diagnostic instruments, such as the Confusion Assessment Method for the Intensive Care Unit (CAM-ICU) (20). Ely, E. Wesley is dedicated to teaching and training healthcare professionals to identify and manage ICU delirium in order to improve clinical care and patients’ prognosis (21). Additionally, their research has provided the scientific foundation for the development of guidelines for the prevention and treatment of ICU delirium, which have been adopted globally.

**Table 1:** The top 10 authors with the largest number of published articles.

Rank	Authors	Publication	Citations
1	Ely, E. Wesley	83	6,763
2	Pandharipande, Pratik P.	35	2,112
3	Girard, Timothy D.	32	1,641
4	Devlin, John W.	26	3,242
5	Thompson, Jennifer L.	23	1,678
6	Jackson, James C.	22	1,452
7	van den boogaard, Mark	22	531
8	Slooter, Arjen J. C.	22	720
9	Brummel, Nathan E.	19	1,301
10	Gao, Sujuan	18	309

### 3.3 Countries and institutions

**Figure 3:**
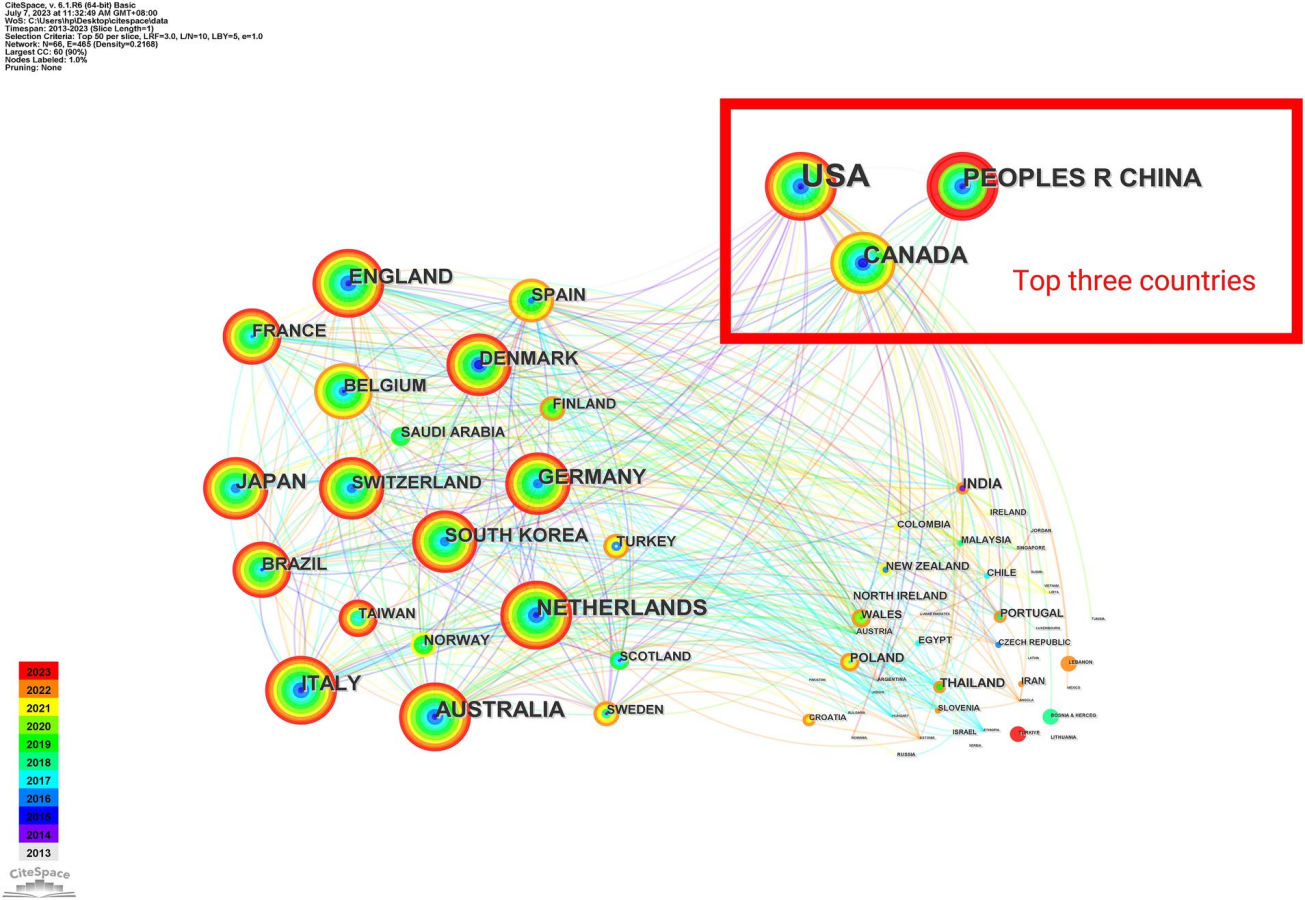
Map of country cooperation networks. Each node corresponds to a country. The size of the node is directly proportional to the quantity of scholarly documents published by that country, with larger nodes signifying a more extensive publication output. The lines interconnecting these nodes signify collaborative efforts, highlighting the interconnectedness and cooperative endeavors among nations in the realm of academic research.

There are 465 connecting lines that show the cooperative relationship between nations, and 66 nodes that represent 66 countries, with the United States, China, and Canada being the top three countries circled in red (Figure 3). This illustrates the dominance of developed nations in the field of international collaboration. The close ties across nations highlight the prevalence of ICU delirium. The red node of China indicates that the publications have a citation or frequency outbreak. In recent years, ICU delirium has received more attention in China, with extensive research on neurobiological mechanisms, treatments, etc.

**Figure 4:**
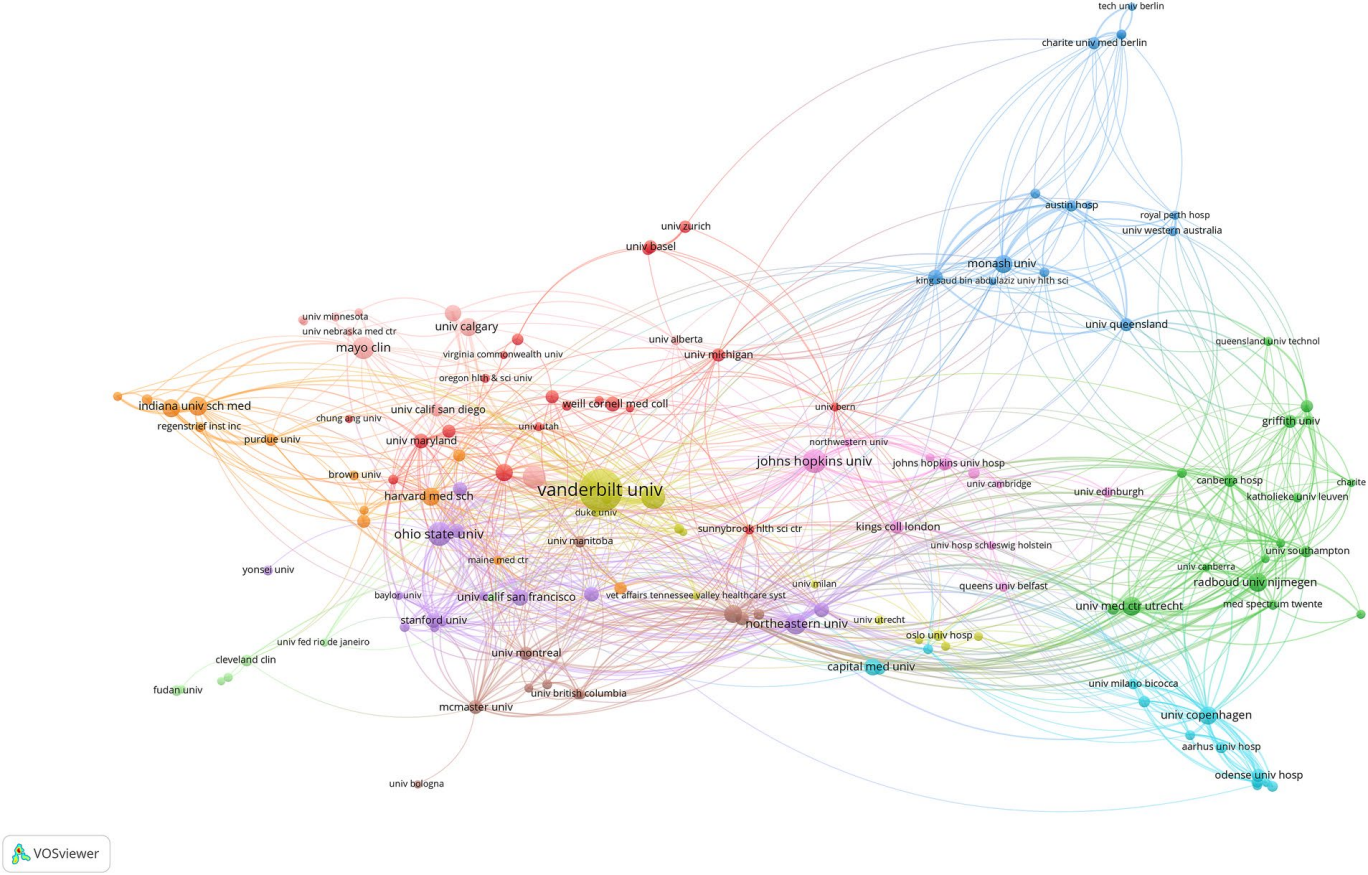
Collaborative map of institutions with publications >5. Each node embodies an institution, with its size indicative of the institution’s publication output—the larger the node, the more prolific the institution. The connections between nodes symbolize the co-authorship relationships, reflecting the collaborative efforts between the respective institutions.

Furthermore, 382 institutions published articles on this subject (Figure 4). Vanderbilt University is one of the institutions with the highest number of relevant articles published (121 in total), and the results cover a wide range of aspects such as the pathogenesis of delirium, diagnostic methods, preventive strategies, and therapeutic measures, and has a high number of citations (9885) (Table 2). This indicates that Vanderbilt University’s research in this field has been widely noticed and recognized, and has made significant contributions to the advancement of ICU delirium research.

**Table 2:** The top 10 institutions with the largest number of published articles.

Rank	Institutions	Countries	Publication	Citations
1	Vanderbilt University	United States	121	9,885
2	Johns Hopkins University	United States	37	1792
3	Ohio State University	United States	37	4,260
4	Tennessee Valley Healthcare System	United States	36	3,596
5	University Pittsburgh	United States	34	1,192
6	Mayo Clinic	United States	32	384
7	Northeastern University	United States	28	1,353
8	University Medical Center Utrecht	Netherlands	24	901
9	Indiana University School of Medicine	United States	24	558
10	Radboud University Nijmegen	Netherlands	23	730

### 3.4 Journals

The top 10 journals published between January 2013 and June 2023 are listed in Table 3, including the country of the journal, the number of articles, and the H-index. Five of them are from the US, four from the United Kingdom, and one from the Netherlands. The distribution of journals shows the impact of research in the field in different countries and regions, while the H-index also reflects the scholarly impact and quality of journals. Critical Care Medicine (*n* = 105) is the journal with the most publications and the highest citation frequency, and it has a high H-index. The premier journal in critical care medicine, Critical Care Medicine Journal, encompasses every aspect of acute and emergency care for patients who are acutely ill or injured. Journal of Critical Care, the second most prolific journal, focuses more on practical aspects of critical care, including clinical care delivery, nursing in critical care, and the improvement of quality of care in critical care units. Articles in these high-impact journals often represent cutting-edge research results and important research directions in the field, and can provide researchers with more valuable information. By analyzing the publication trends of journals, researchers can identify the hotspots of journals, adjust research direction and content, and improve the success rate of submissions.

**Table 3:** The top 10 journals with the largest number of published articles.

Rank	Journals	Countries	Publications	H-index
1	Critical care medicine	United States	105	294
2	Journal of critical care	Netherlands	80	96
3	BMJ open	United Kingdom	46	139
4	Intensive and critical care nursing	United States	41	63
5	Nursing in critical care	United Kingdom	35	49
6	Plos one	United States	33	404
7	American journal of critical care	United States	30	87
8	Critical care	United Kingdom	29	200
9	Trials	United Kingdom	29	91
10	Critical care nurse	United States	26	48

### 3.5 Keywords

**Figure 5:**
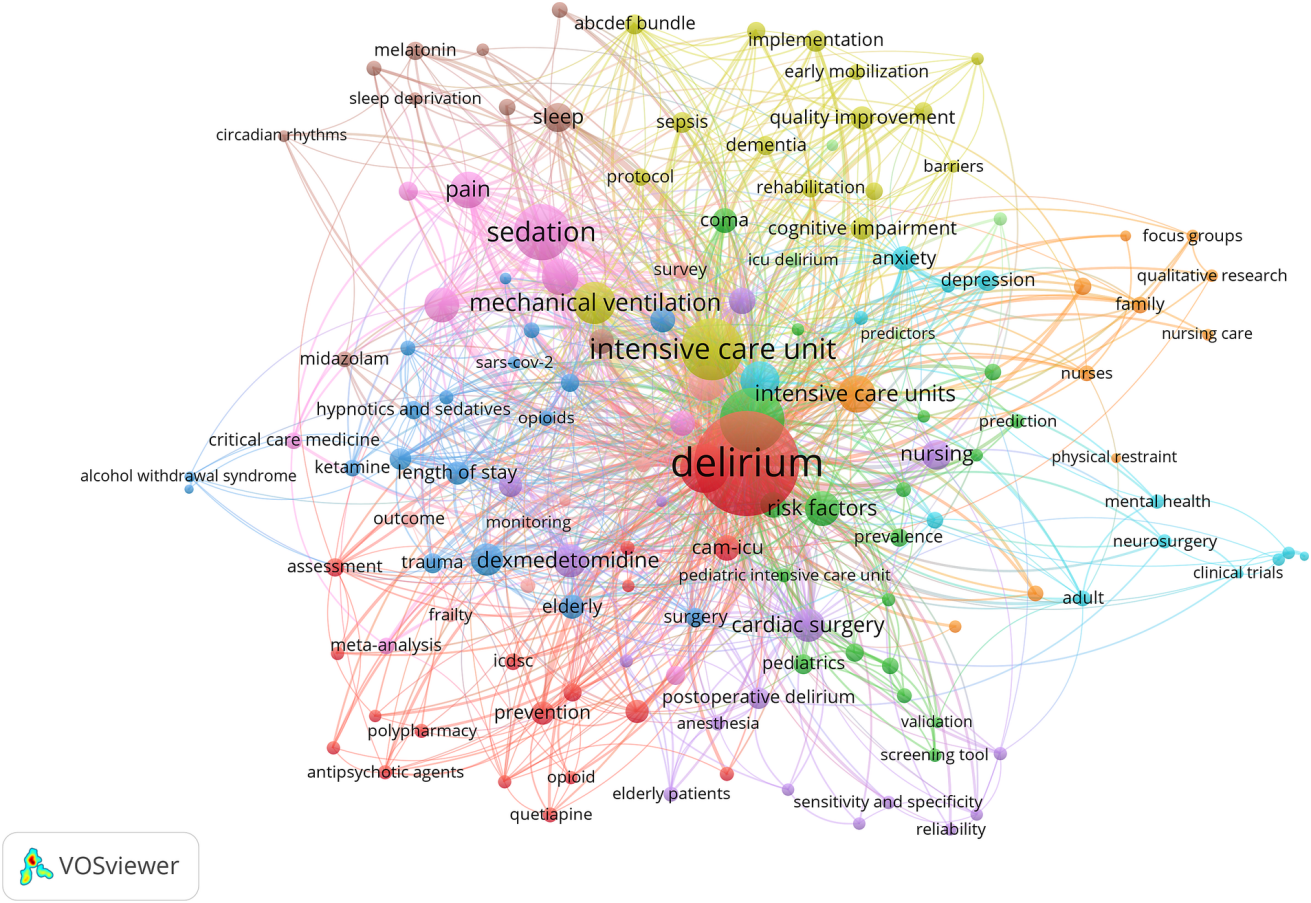
Co-occurrence network diagram of keywords that appear more than five times. Each node corresponds to a specific keyword, with its size reflecting the keyword’s frequency across various time periods. The links connecting these nodes signify the co-occurrence frequency of the associated keywords, indicating how often they appear together in publications.

Keywords can be utilized in bibliometrics to reflect research trends and hotspots in the field (22). Using VOSviewer, 143 terms with a frequency of at least five times were displayed. Each node in Figure 5 represents a distinct keyword, and the size of the node indicates how frequently the keyword occurs over time. The larger the node, the more frequently the keyword occurs, suggesting that the keyword is related to the research topic in the area that is receiving the most attention and could potentially be the hot spot direction of the study. The co-occurrence frequency of the keywords is the frequency of simultaneous appearance in the same literature. It is indicated by the line connecting the nodes, and the thicker the line, the stronger the association between the two keywords. The highly important terms were divided into 11 clusters, each denoted by a distinct color. “Delirium” is the biggest node, and terms like “antipsychotic agents,” “polypharmacy,” and “quetiapine” are linked to it. This implies a connection between the onset of delirium and the usage of various antipsychotics. The term “mechanical ventilation” is shown next to “intensive care unit,” suggesting that the use of mechanical ventilation increases the likelihood of developing delirium (23–25).

**Figure 6:**
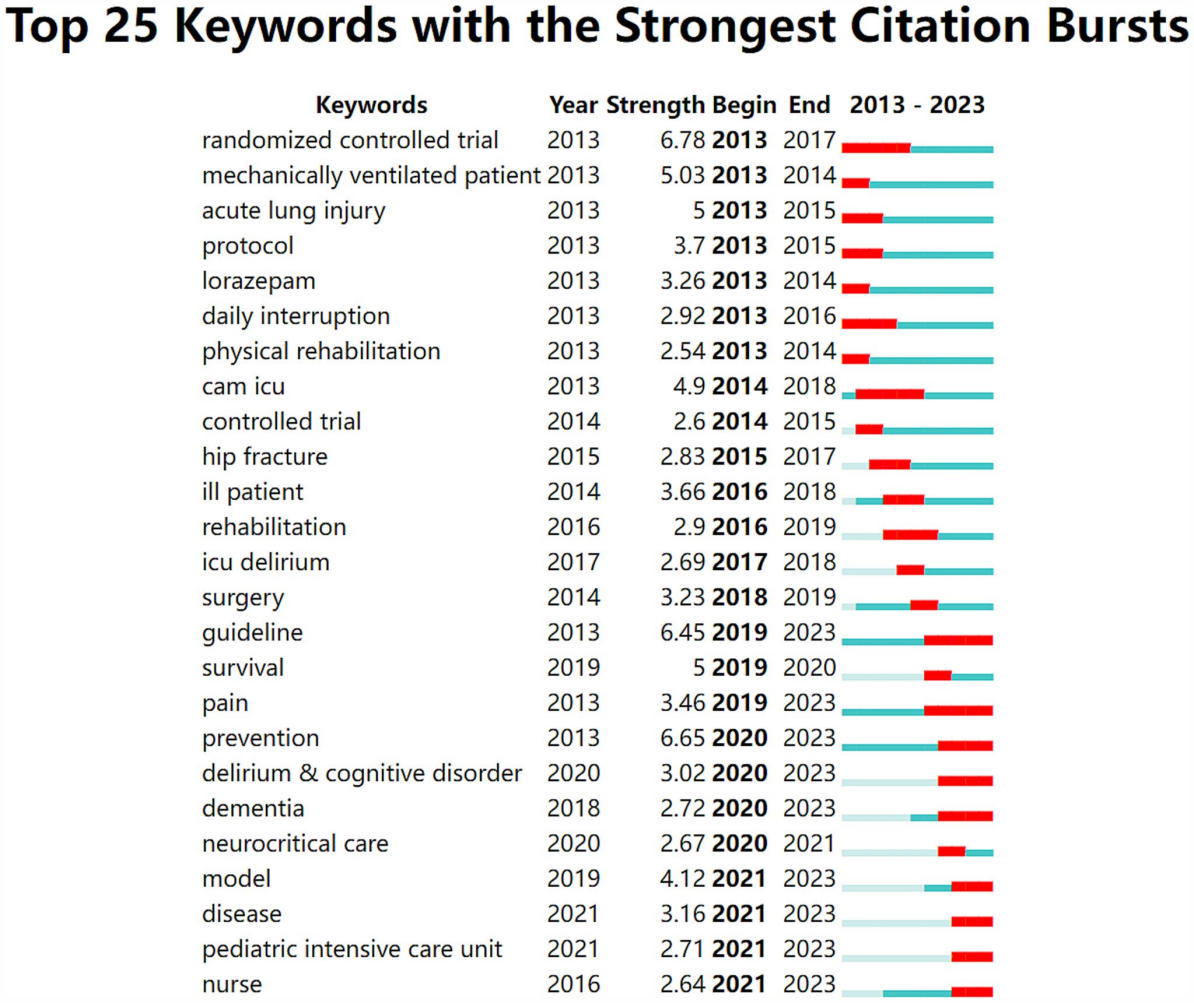
The blue line delineates the comprehensive timeline of the publication period under review, spanning from 2013 to 2023. The red line highlights a particular time frame associated with a burst word, which has experienced a significant surge in usage during a specific interval.

Furthermore this study also applied CiteSpace to mutate keywords in the selected 1178 articles and obtained 25 mutated keywords as shown in Figure 6. They are principally classified as follows: research methodology (randomized controlled trial; protocol; controlled trial; model; guidelines), custodial ward (ICU; neurocritical care; pediatric intensive care unit), predisposing factor (mechanically ventilated patient; acute lung injury; hip fracture; ICU delirium; surgery; pain; delirium and cognitive disorder; dementia; disease; ill patient), prognosis and rehabilitation (lorazepam; daily interruption; physical rehabilitation; rehabilitation; survival; prevention; nurse). Burst keywords are words frequently cited over time which can reflect research trends (26). Figure 6 shows the entire period in blue with the citation burst duration in red. The most intensively cited keywords were randomized controlled trial (strength 6.78 time span 2013-2017) prevention (strength 6.65 time span 2020-2023) guideline (strength 6.45 time span 2019-2023) mechanically ventilated patient (strength 5.03 time span 2013-2014) acute lung injury (strength 5 time span 2013-2015). Early researchers used a large number of randomized controlled trials to test hypotheses and interventions laying the foundation for subsequent research. During the period from 2013 to 2015 “mechanically ventilated patients” and “acute lung injury” were the keywords with high citation intensity indicating that the research focus at that time was on the complications closely related to ICU delirium. In recent years researchers have taken a multi-pronged approach to preventing delirium by following a guiding route that involves early identification of risk factors optimization of treatment regimens and enhancement of healthcare training. In the future researchers can delve deeper into the complex network of interactions between risk factors develop more accurate risk prediction models and further explore the research areas represented by each keyword appearing in the "citation burst" during the research progress.

### 3.6 Co-cited reference

**Figure 7:**
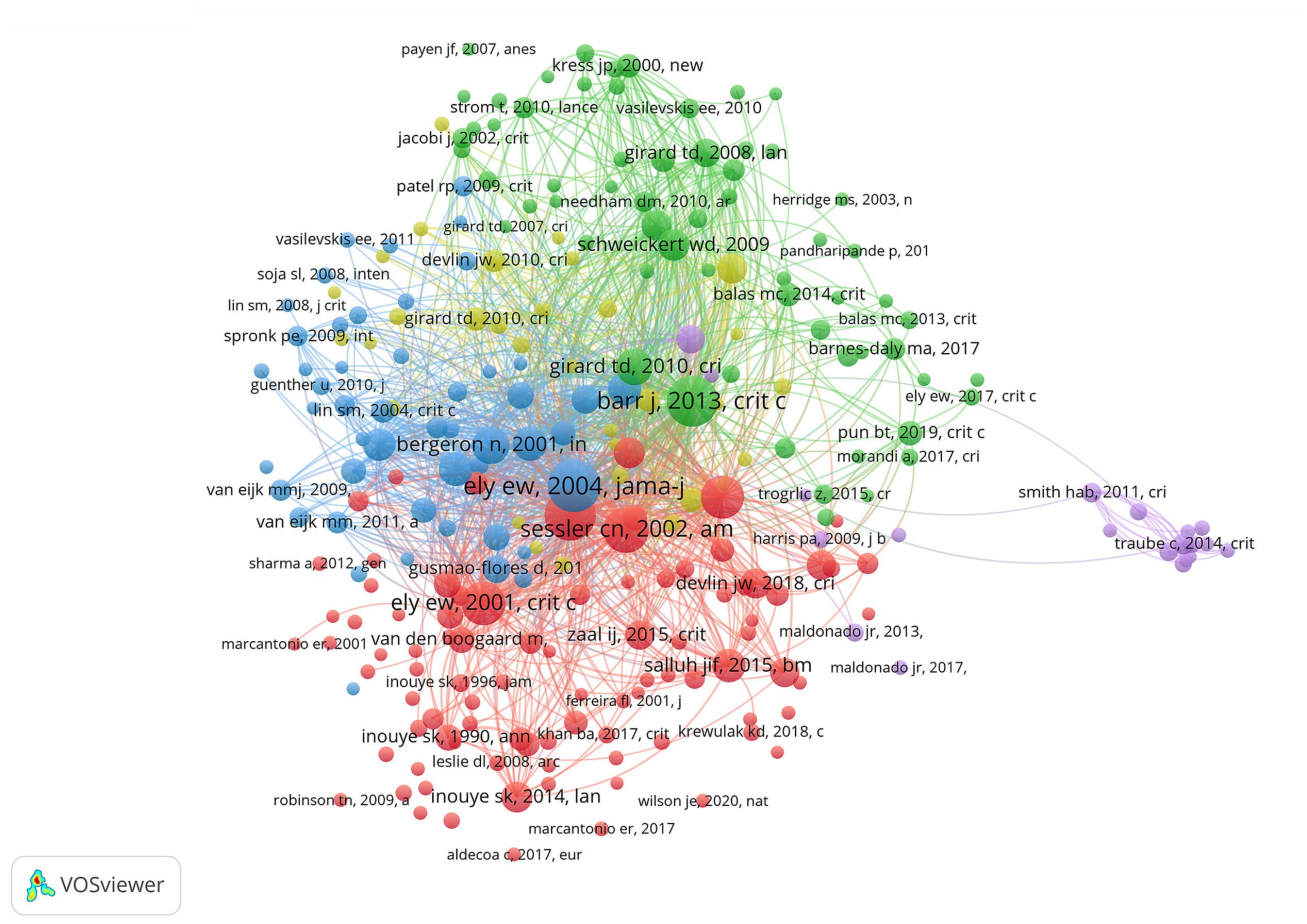
Co-citation analysis network diagram for references with more than 50 citations. The size of each node is proportional to the frequency of citations it has received. Larger nodes indicate that the associated research has garnered a higher number of citations. The connections between nodes illustrate the citation relationships, mapping out the academic lineage and scholarly influence among various studies.

The term “co-cited references” refers to references that have been cited by more than one article. VOSviewer software was used to map the network among document references (Figure 7). A total of 253 documents had more than 20 citations. Larger nodes such as “Ely EW, 2004, Jama-j”, “Barr J, 2013, crit c”, and “Ely EW, 2001, Jama-j” belong to the key references in the field, and those with strong correlation are in the same color category. Based on the observation and data analysis of numerous ICU patients, it was discovered that delirium not only lengthens the time that patients require mechanical ventilation but also considerably raises their death rate (Ely Ew, 2004, Jama-j). This finding prompted clinicians to pay close attention to ICU delirium, and numerous studies began to concentrate on this subject in an effort to identify effective interventions, such as early rehabilitation exercises and optimization of the sedation strategy, to lower the incidence of delirium, thereby shortening the duration of mechanical ventilation and reducing the risk of patient death.

**Figure 8:**
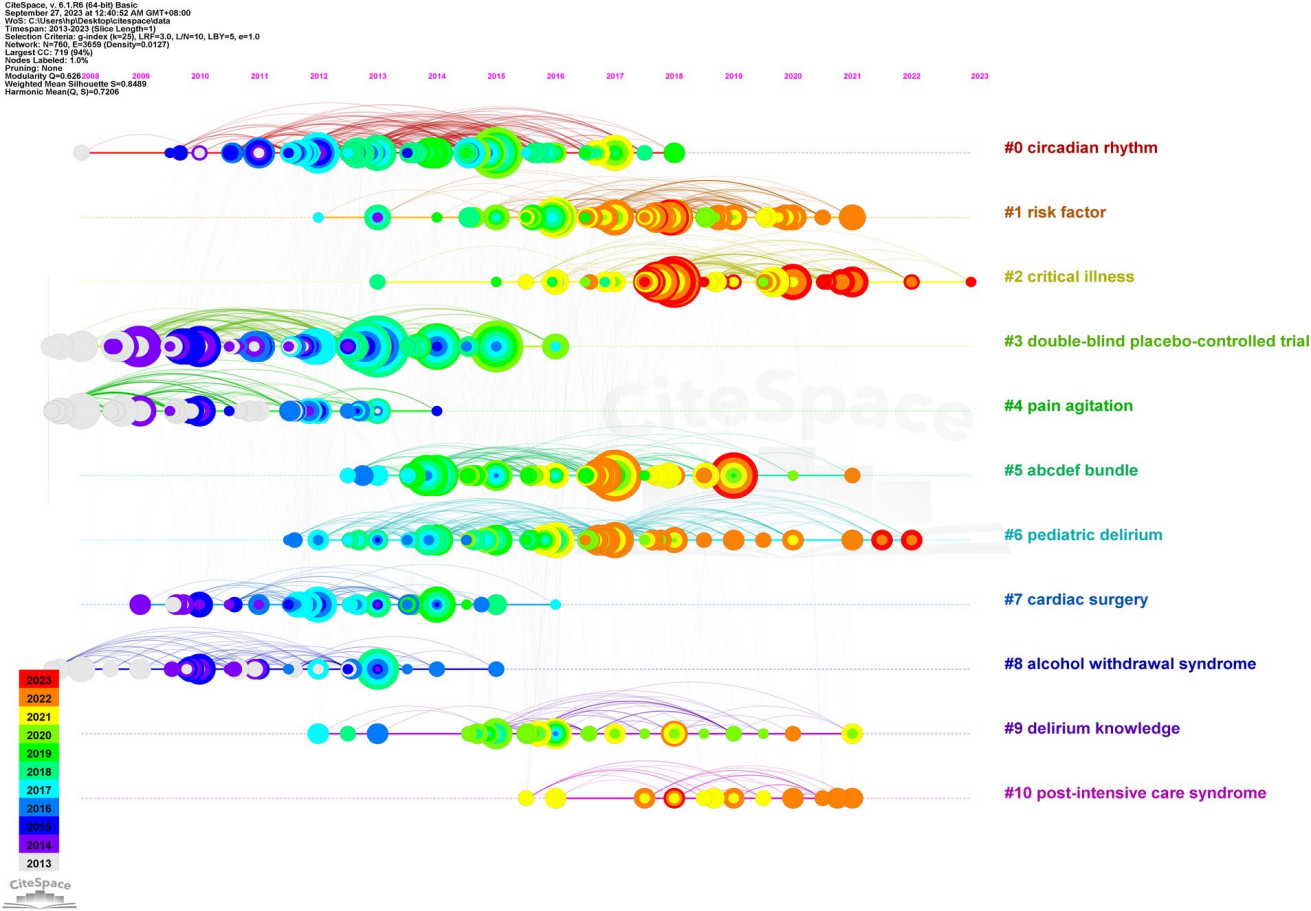
Time map of the best 11 co-cited network clusters in the ICU delirium references. Each cluster captures the research hotspots of a particular era, as delineated by the timeline.

In addition, 1,176 documents from the database were clustered using Citespace software to extract the noun terms in the titles of the documents, and to draw a time plot of the clusters according to the time and the size of the clusters. Finally, eleven clusters were obtained (Figure 8), including cluster 0 (circular rhythm), cluster 1 (risk factor), cluster 2 (critical illness), cluster 3 (double-blind placebo-controlled trial), cluster 4 (pain agitation), cluster 5 (ABCDEF bundle), cluster 6 (pediatric delirium), cluster 7 (cardiac surgery), cluster 8 (alcoholic withdrawal syndrome), cluster 9 (delirium knowledge), and cluster 10 (post-intensive care syndrome). It can also be seen that clusters 2 and 6 are still undergoing continuous evolution.

## 4 Discussion

According to the WOS database, a total of 1,176 publications about ICU delirium were published between 2013 and June 2023. Except for 2019, the number of publications showed an upward trend each year, with the highest number in 2021, indicating a growing interest in ICU delirium among scientists. Ely, E. Wesley, the scientist with the most publications, has focused his research on developing better treatments and improving outcomes for critically ill patients with ICU-acquired encephalopathies. This not only advances the understanding of ICU delirium but also reflects its dynamic nature. The United States is the nation with the most publications and also has the most journals and organizations, demonstrating its strong scientific foundation. In terms of journals, scientists might consider Critical Care Medicine as their target because it has the greatest number of published articles and a comparatively high H-index. To comprehend ICU delirium and guide future research, we have broadly grouped ICU delirium into several categories, including risk factors, clinical presentation, diagnosis, prevention and treatment, based on emerging keywords and literature clustering.

### 4.1 Risk factors

Up to 80% of individuals in the ICU experience delirium, which typically manifests as neurologic impairment (3, 11). There are two types of risk factors for delirium in the ICU: susceptibility factors and precipitating factors. The susceptibility factors of ICU delirium include: (1) older adults (aged 65 and older) (27–30), (2) Pre-existing neurological disorders (such as dementia, cognitive impairment, depression, etc.) (31–34), (3) cancer (35), (4) patients undergoing major surgery, (5) multiple comorbidities (electrolyte disorders, severe hypoproteinemia, etc.) (36, 37), (6) drug and tobacco withdrawal (28), (7) sensory impairments (e.g., vision, hearing), and functional impairments (i.e., reduced activities of daily living) (38–40). Exposure of patients with susceptibility factors to precipitating factors will lead to the development of delirium. The following are the precipitating factors for the ICU delirium: (1) Drug use: anticholinergics (41), opioids (42), benzodiazepines (38, 43), psychoactive drugs (28). (2) Surgery: hip surgery in the elderly (44), prolonged extracorporeal circulation in cardiovascular surgery (45), (3) Environment: mechanical ventilation (20, 42), physical restraints, catheters, and various drains (3, 46), circadian rhythm (47), (4) Comorbidities: infections, multiorgan failure following surgery (48), anemia, malnutrition, etc. (49). Within the clinical setting, healthcare professionals ought to evaluate the delirium risk of patients more thoroughly. To lower the incidence of delirium, preventive measures should be taken beforehand and surveillance need to be enhanced for patients with risk factors.

### 4.2 Clinical presentation

Delirium typically occurs one or two days after the patient is admitted to ICU and lasts for an average of three days before improvement (20, 42, 50–53). Depending on the activity level during the episode, delirium can be categorized as hyperactive, hypoactive, or mixed delirium (54). Hyperactive delirium is characterized by restlessness, irritability, excitement, increased volume, hallucinations, etc., which is easy to detect. Clinically, such patients may be at an increased risk of self-injury and present a significant challenge to caregivers. Reduced activity, such as drowsiness, silence, lowered level of awareness and reduced spoken exchange, is a hallmark of hypoactive delirium, which is commonly seen in the elderly. Since hypoactive delirium is difficult to identify (1, 55), it may lead to a higher incidence of complications, such as lung infections and thrombosis. Mixed delirium may exhibit alternating features of hyperactive and hypoactive delirium within a brief period. These symptoms change with the course of the disease. In the ICU, hypoactive and mixed delirium account for more than 90% of patients (7, 56). Therefore, improving the ability of healthcare professionals to recognize delirium is crucial.

### 4.3 Diagnosis

Timely detection, early diagnosis and treatment of delirium are critical to improving patient outcomes (57, 58). Diagnostic and Statistical Manual of Mental Disorders (DSM-5) is considered as the gold standard of diagnosis of delirium (54). The features of DSM-5 include: (1) Deficits in awareness and attention are characterized by a decline in orientation to the surroundings and a reduction in the capacity to focus, direct, sustain, and shift focus; (2) Attention and consciousness deficits that progress over hours or days, with fluctuating manifestations of the condition; (3) Being accompanied by cognitive deficits in memory, orientation, language, visuospatial awareness, or sensorimotor perception; (4) Not being able to be explained by other pre-existing neurologic cognitive deficits; and (5) evidence associated with the onset of delirium, such as drug intoxication or withdrawal, exposure to toxins, etc. Other possible symptoms include altered arousal, disorientation, confusion, improper words or behavior, disrupted circadian rhythm, psychomotor abnormalities and hallucinations.

Even though the DSM-5 is the most reliable tool for delirium diagnosis, its implementation requires high professional standards and is time-consuming and laborious, making its application in clinical work challenging. There are numerous patients in critical, complex, and rapidly evolving condition. Therefore, additional delirium assessment instruments have been developed in clinical practice, taking into account the unique characteristics of ICU patients, such as CAM-ICU, Intensive Care Delirium Screening Checklist (ICDSC), Nurses’ Delirium Screening Scale (Nu-DESC), Confusion, Tolerance, and Delirium (CTD), Nursing Evaluation of Elderly Comatose or Handicapped Mental state (NEECHAM), and Delirium Detection Score (DDS) (3, 59, 60). Among them, Confusion Assessment Method (CAM) is the most widely used, which has four clinical features: (1) acute change and a varying path; (2) inattentiveness; (3) disorganized thinking; and (4) altered awareness. If one of features 3 or 4 as well as criteria 1 and 2 are met, delirium can be diagnosed (61). CAM-ICU as well as 3D-CAM are further optimizations of CAM, which have a high degree of utility, sensitivity, and specificity (62). CAM-ICU can be used in patients who are using a mechanical ventilation system and unable to speak (57). The ICDSC consists of eight items (63), which examine altered awareness, inattention, psychomotor agitation or retardation, hallucinations, delusions or psychosis, inappropriate speech or mood, sleep–wake/cycle disruptions, and symptom fluctuations. The sensitivity and specificity of various assessment instruments vary. Although the CAM-ICU and ICDSC are the most precise and trustworthy evaluation instruments for delirium in adult ICU patients, a suitable one should be chosen based on patients’ unique circumstances and hospitals’ actual settings (64–74).

### 4.4 Prevention

Early detection of delirium is critical to preventing its onset (75), and the cornerstone of ICU delirium care is nonpharmacologic prevention. Non-pharmacologic prevention may reduce the incidence of delirium by 50% (76), including (1) Early rehabilitation exercises: Early rehabilitation activities should be personalized and tailored to the patient’s specific condition and physical status. Simple physical exercises can commence as soon as the patient’s sedation level permits; when the patient’s condition stabilizes, the intensity and complexity of the activities can be gradually increased (77). It has been shown that early rehabilitative exercise reduces delirium duration by 2 day and is safer (78). (2) Psychosocial support: ICU delirium can also be effectively reduced by healthcare professionals and patients’ family members by actively asking patients about their needs, placing familiar objects around them, and giving appropriate psychological support (79, 80). (3) Improve sleep quality: In a study by Al-Aama et al. (81), melatonin was found to improve the quality of sleep and decrease the risk of delirium in elderly patients. Zaal (82) also revealed that improving the surroundings by reducing noise and increasing daylight exposure reduced the duration of delirium. (4) Avoid drugs such as antihistamines (e.g., diphenhydramine, methotrexate) (83, 84), benzodiazepine (e.g., lorazepam and midazolam) (53, 85, 86) tricyclic anxiety medications (e.g., amitriptyline, promethazine) (87), and other anticholinergic drugs (e.g., atropine, scopolamine) (41, 88). The creation of a delirium prevention team can help reduce postoperative delirium in terms of stress management, cognitive function improvement, and delirium risk factors assessment (89).

### 4.5 Treatment

Medication and psychological / behavioral intervention are the primary treatments for delirium (90). Dexmedetomidine is a centrally active agonist of the alpha-2 adrenergic receptor with sedative, analgesic, and anxiety-relieving effects with no effect on patients’ cognitive performance. Research reveals that administering dexmedetomidine to ICU patients undergoing mechanical ventilation dramatically shortens the duration of delirium and expedites extubation (91). Postoperative intravenous administration of acetaminophen combined with dexmedetomidine or isoproterenol decreases the incidence of delirium (92). The incidence of delirium might be decreased by using exogenous melatonin. Nevertheless, the effect of melatonin has been inconsistent in clinical pilot studies. There was no statistically significant difference in the incidence of delirium in individuals with hip fractures between the melatonin and placebo groups; however, in an internal medicine ward trial involving elderly patients, melatonin treatment significantly decreased the risk of delirium (81, 93). Statins inhibit cholesterol synthesis and have anti-inflammatory and neuroprotective effects that may benefit patients with delirium (88).

The concept of clustering refers to assembling a series of effective treatment and care measures for a disease based on evidence-based medicine to improve the overall clinical outcome. For mechanically ventilated patients in ICU, there is currently a clustered care plan that includes the following: (1) assessment, prevention, and control of pain; (2) conducting spontaneous respiratory and arousal tests; (3) analgesic and sedation strategies; (4) assessment, prevention, and management of delirium; (5) early activity and exercise; and (6) family involvement and empowerment (94). By reducing the likelihood of delirium and the need for mechanical ventilation in the critical care unit, the above bundle improves patient outcomes (95–97). In addition, delirium duration can also be shortened by family-oriented psychotherapy, such as familiar surroundings and the company and comfort of loved ones. Improving the surrounding environment by reducing noise, music therapy (98) and increasing daylight exposure can shorten delirium duration (99–101). Nonpharmacologic preventive strategies, like improving the sleep environment and using drugs appropriately, can lower the risk of morbidity and improve prognosis. These strategies offer new options for clinical treatment as researchers better understand delirium. Research has been done to create customized therapy regimens to lower the incidence of delirium from a variety of angles depending on the presence of risk factors, intraoperative drugs, and other variables (102). This study gives physicians a thorough reference for managing and preventing postoperative delirium, which can help raise awareness of the condition, encourage better clinical practice prevention strategies, enhance patient outcomes, and cut down on medical resource waste.

### 4.6 Limitations

There are some potential limitations in the current study. Firstly, this study only incorporated English-language literature published after 2013, restricted the publication type to “Article,” and excluded academic reports, conference papers, and other materials. Consequently, significant research findings from non-English-speaking nations may have been overlooked, introducing a language bias to the study. Secondly, the data utilized in this study was sourced from the WOS database, which is a fairly reliable information source. Although the WOS database furnished our study with highly dependable data, it might not comprehensively cover all the pertinent literature. It is recommended that future research consider multilingual literature and leverage additional databases such as Scopus to enhance the study’s accuracy and comprehensiveness.

## 5 Conclusion

The current study reflects the research hotspots, trends, and research directions of ICU delirium over the past ten years. Per keywords analysis, the risk factors, clinical presentation, diagnosis, prevention and treatment of ICU delirium were reviewed. These findings will improve understanding of the current status of ICU delirium research and offer valuable information for further investigation.

## Data Availability

The original contributions presented in the study are included in the article/supplementary material, further inquiries can be directed to the corresponding author/s.
